# A RUBY Reporter for Efficient Banana Transformation and Development of Betalain-Rich *Musa* Germplasm

**DOI:** 10.3390/ijms26167805

**Published:** 2025-08-13

**Authors:** Weidi He, Huoqing Huang, Shuxian Wang, Dalin Wang, Yanling Xie, Chunhua Hu

**Affiliations:** 1 Institute of Fruit Tree Research, Guangdong Academy of Agricultural Sciences, Key Laboratory of South Subtropical Fruit Biology and Genetic Research Utilization, Ministry of Agriculture and Rural Affairs, Guangdong Provincial Key Laboratory of Tropical and Subtropical Fruit Tree Research, Guangzhou 510640, China; heweidi89@163.com (W.H.); hqhuang07@163.com (H.H.); wangshu614@126.com (S.W.); 13265141121@163.com (D.W.); xieyanling18@163.com (Y.X.); 2 College of Life Sciences, South China Agricultural University, Guangzhou 510642, China

**Keywords:** banana, betalain, genetic transformation, RUBY

## Abstract

Bananas are economically important crops valued for both their nutritional and dietary uses. However, the global banana industry suffers from a narrow base dominated by a single variety. Developing novel varieties enriched in health-promoting compounds such as betalains can help diversify banana germplasm and meet evolving consumer demands. In this study, the RUBY reporter system was employed to produce betalain-rich bananas via stable and transient genetic transformations. Transient transformation by injecting 3 mL of *Agrobacterium* suspension into immature fruits produced vivid red-purple pulp containing up to 1.78 mg/g of betalains. For stable transformation, embryonic cell suspensions expressing RUBY exhibited a red-purple coloration after the first screening, reducing the selection period from 45 to 15 days. These findings demonstrate that RUBY is a reliable visual reporter for efficient screening and can be used to develop nutritionally enhanced bananas.

## 1. Introduction

Bananas (*Musa* spp.) are a staple food in tropical or subtropical regions and a major food source for millions, particularly in Africa and South America, where they provide up to 25–35% of the daily nutrient intake [1]. However, banana cultivars are highly susceptible to Fusarium wilt, a disease caused by *Fusarium oxysporum* f. sp. *cubense*, especially tropical race 4 (*Foc* TR4) [2]. Due to a lack of resistant resources and global trading, *Foc* TR4 has a devastating economic impact on the banana industry and has spread rapidly across the globe. This pathogen has been discovered in many countries where banana is a staple food, such as Mozambique in 2013, Colombia in 2019, and Peru in 2021 [3]. The traditional strategies failed to manage Fusarium wilt. For example, chemical controls are inefficient because the fungi are soil-borne, making them difficult to kill. In addition, there is still a long way to go until biological controls achieve satisfactory results in the field. Therefore, disease-resistance breeding provides a promising strategy for *Foc* TR4 management. Molecular breeding is one of the most important and effective approaches for disease-resistance breeding. This approach requires additional research on visible genetic improvement biotechnology of banana.

It is estimated that there are more than 500 banana varieties, but the main commercial varieties are Cavendish (AAA group), East African Highland Banana (AAA group), plantain (AAB) and Pisang Awak (ABB group). Global banana production reaches approximately 139.3 million tons annually [4], with the Cavendish group in the banana-producing countries accounting for nearly half of the total yield, including Gran Nain and Dwarf Cavendish [5]. Cavendish cultivars are often susceptible to *Foc* TR4, but they are often resistant against *Foc* race 1. East African Highland Banana and plantains are distributed across Africa and Latin America, such as Mozambique, Colombia, Uganda, and Peru. Production of plantain and cooking banana has reached approximately 44.4 million tons in recent years [4]. Pisang Awak is mainly favored by countries in Southeast Asia, such as China and India. Notably, the Pisang Awak group, including Karpuravalli [6] and Guangfen No. 1 [7], is often susceptible to race 1 and race 4 strains. However, the industry’s dependence on this single variety limits its genetic diversity and ability to meet diverse consumer preferences. This monoculture plantation is also an important reason for the rapid spread of *Foc* TR4. To change this situation, introducing disease-resistant genes might contribute to its resistance to the pathogens. For example, a *MamRGA2* resistance gene from a *Foc*-TR4-resistant wild banana was transformed into Cavendish cultivars, and the transgenic plants demonstrated high resistance against Fusarium wilt [8,9]. In addition, knocking out the negative regulator of plant defense can also increase plant resistance. For example, MusaDMR6 [10] or MusaENOD3 [11] knocked out using the CRISPR/Cas 9 system could confer resistance to BXW. However, previous identification of the positive transgenic plants requires complicated PCR and sequencing during the transformation process. Therefore, based on the diverse demands, developing bananas with enhanced visual and nutritional traits is a key strategy for addressing this issue.

Betalains are natural, water-soluble red-purple pigments found in Caryophyllales plants, such as beets, Swiss chard, and pitaya. Owing to their vibrant color, antioxidant properties, and potential health benefits, betalains are widely used as natural food colorants and are under investigation for functional food applications [12,13]. Since edible sources of betalains are limited, biofortification has emerged as a promising approach to increase their availability in commonly consumed crops.

The RUBY reporter system is a synthetic betalain biosynthesis pathway enabling visible red pigmentation in transformed plants [14,15]. This pathway only requires three enzymatic steps: hydroxylation of tyrosine by CYP76AD1 to produce L-3,4-dihydroxyphenylalanine (L-DOPA), conversion of L-DOPA to cyclo-DOPA and betalamic acid (via CYP76AD1 and DODA), and final glucosylation of betanidin by glucosyltransferases to form betalains [16]. RUBY has been successfully used to engineer betalain production in various crops, including rice [14,17], tomato [18,19], maize [19], and carrot [20], highlighting its utility as both a reporter and a biofortification tool.

Notably, bananas are naturally rich in L-DOPA, a key precursor in the betalain biosynthetic pathway, with Cavendish pulp containing up to 4.02 mg/100 g [21]. This suggests that bananas are well suited for betalain biofortification. Prior bioengineering efforts have produced nutrient-enriched bananas, such as vitamin A-rich “Golden Banana” [22] and iron-rich varieties [23]. However, to date, no studies have reported the creation of betalain-rich banana germplasm.

In this study, we used the RUBY system to generate banana seedlings and fruits with high betalain content via stable and transient transformation. This work not only demonstrates a method to enhance the visual appeal and nutritional value of bananas but also introduces RUBY as an effective visual marker to improve the screening efficiency of banana genetic transformation.

## 2. Results

### 2.1. RUBY Drives Betalain Synthesis in Nicotiana Benthamiana Leaves and Banana Fruit

**Figure 1:**
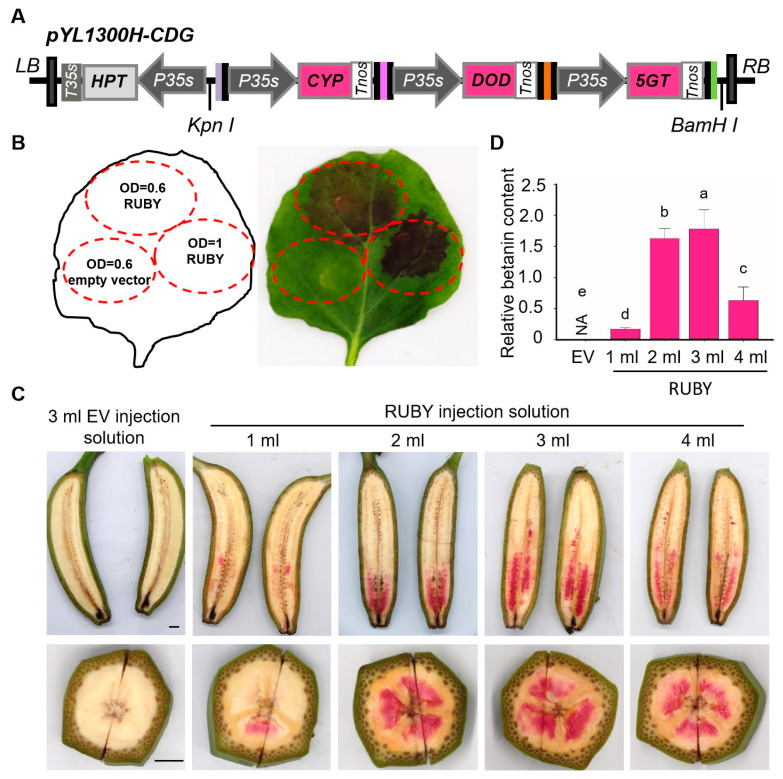
Engineering betalain biosynthesis in *N. benthamiana* leaves and banana fruits. (**A**) Schematic diagram of the pYL1300H-CDG vector. Three betalain biosynthesis genes [BvCYP76AD1S (C), BvDODA1S (D), and cDOPA5GT (G)] were assembled into the pYL1300H-UNiE binary vector. (**B**) Phenotype of *N. benthamiana* leaves after Agrobacterium-mediated transient expression. EV: empty vector (OD_600_ = 0.6, bottom left); RUBY vector at OD_600_ = 0.6 (top) and OD_600_ = 1.0 (right). (**C**) Phenotype of banana fruit injected with RUBY vector (1–4 mL); EV injected with 3 mL was used as the control (left). Bar = 1 cm. (**D**) Quantification of betalains in banana pulp. EV was used as the negative control. Data are presented as means ± SDs (one-way analysis of variance, *p* < 0.05, *n* = 3). Different letters above the columns indicate a significant difference.

To assess betalain production, RUBY plasmid was transiently expressed in *N. benthamiana* leaves (Figure 1A). Three days post-infiltration, red-purple spots were observed in RUBY-infiltrated leaves but not in the control (Figure 1B). Increasing the *Agrobacterium* OD_600_ from 0.6 to 1.0 enhanced pigment intensity (Figure 1B). Transient expression was also tested in banana fruits. As shown in Figure 1C, red-purple spots appeared in the pulp 4 days after RUBY injection. Increasing the injection volume from 1 mL to 3 mL caused the colored region to expand from the top to the middle of the fruit fingers. However, at 4 mL, the pigmentation spread further, but the colored area decreased, likely due to tissue damage or saturation. Betalain levels in the colored banana pulp were quantified (Figure 1D). The highest content, 1.78 mg/g, was observed with a 3 mL injection volume, suggesting this as the optimal dosage for transient expression.

### 2.2. RUBY Synthesizes Betalains in Banana Plants

**Figure 2:**
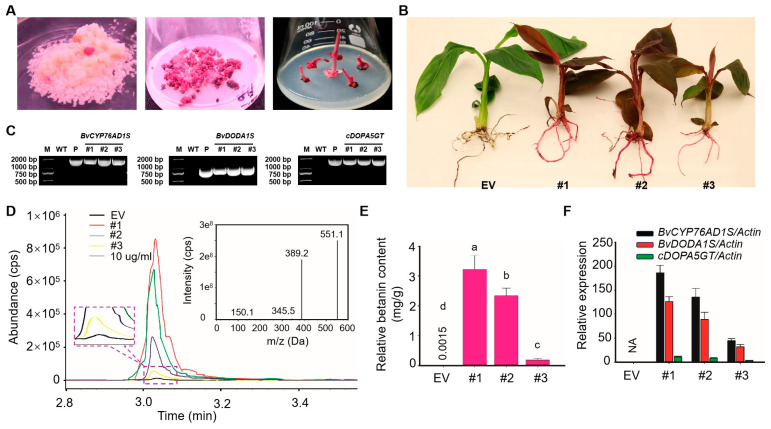
Phenotypic observation and identification of red-purple transgenic bananas. (**A**) Growth stages of Agrobacterium-mediated transformation: embryo culture, somatic embryo germination, and rooting. (**B**) Phenotypes of EV control and RUBY transgenic plants. (**C**) PCR amplification of BvCYP76AD1S, BvDODA1S, and cDOPA5GT. (**D**) LC-MS chromatograms of control and transgenic plants. (**E**) Quantification of betalain content. (**F**) Relative expression levels of betalain biosynthetic genes. Data are presented as means ± SDs; different letters above the columns indicate a significant difference (one-way analysis of variance, *p* < 0.05, *n* = 3).

Following the initial hygromycin resistance screening (~15 days), transformed ECSs expressing RUBY developed into visible red-purple granular somatic embryos. These embryos were transferred to regeneration medium, where bud formation occurred over the course of one month (Figure 2A). The red-purple regenerated buds were individually expanded and subsequently placed in rooting medium for one month of cultivation. Genomic DNA was extracted from transgenic lines and subjected to PCR analysis. Target amplicons of 1491 bp, 825 bp, and 1511 bp were successfully amplified in all three red-purple lines, while no amplification was detected in the control (Figure 2C). The transgenic plants were grown in a greenhouse and displayed normal growth; however, red-purple pigmentation intensity varied among lines, with line #1 exhibiting the most vivid coloration (Figure 2B).

To quantify betalain accumulation, liquid chromatography–mass spectrometry (LC-MS) was performed. The retention time of standard betalains was recorded at 3.03 min with a mass-to-charge ratio of 551.1 Da (Figure 2D). As shown in Figure 2E, betalain concentration in control leaf tissue was 1.50 ug/g, while the levels in transgenic lines reached 3.23 mg/g, 2.34 mg/g, and 0.18 mg/g. Relative expression levels of BvCYP76AD1S, BvDODA1S, and cDOPA5GT were quantified by qRT-PCR. These genes were not expressed in control plants, while variable expression was observed in transgenic lines. A positive correlation was noted between pigmentation intensity and gene expression, with line #1 exhibiting the highest expression across all three genes (Figure 2F).

### 2.3. Betalain Accumulation Slightly Affects Banana Plant Growth

**Figure 3:**
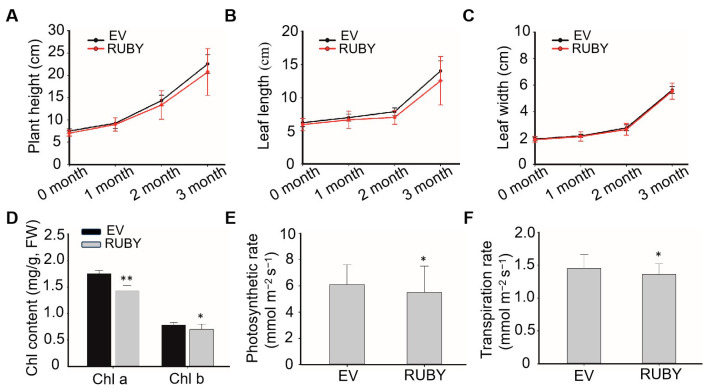
The effect of betalain synthesis on agronomic traits of transgenic banana plants. (**A**) Plant height, (**B**) leaf length, (**C**) leaf width of RUBY (line #1) and EV transgenic plants were measured over three months. Plant height is measured from the above-ground pseudostem to the heart leaf, leaf length is measured from the petiole to the tip, and leaf width is measured at the widest part of the leaf. (**D**) Chl content, (**E**) photosynthetic rate and (**F**) transpiration rate of RUBY (line #1) and EV transgenic plants were measured on the third month after transplanting. Data are presented as means ± SDs (two-tailed Student’s *t*-test, * *p* < 0.05, ** *p* < 0.01, *n* = 6).

To assess the physiological impact of betalain accumulation, plant height, leaf length, and leaf width were measured over three months. In the third month, chlorophyll content, the net photosynthetic rate, and the transpiration rate were also evaluated. As shown in Figure 3A,B, transgenic plants exhibited slightly reduced plant height and leaf length relative to controls, while leaf width remained comparable between groups (Figure 3C). Furthermore, chlorophyll content, the net photosynthetic rate, and the transpiration rate were all lower in transgenic plants, with a significant reduction in chlorophyll content (Figure 3D–F). These findings suggest that betalain biosynthesis exerts a modest inhibitory effect on banana plant growth.

## 3. Discussion

### 3.1. Betalains Enrich the Appearance and Nutritional Value of Bananas

Betalains are important pigments widely used as food colorants and are known for their health-promoting properties, including antioxidant, antitumor, and antimalarial activities. Natural sources of edible betalains are limited, primarily found in plants such as beet, amaranth, and pitaya. Therefore, enhancing betalain content through biofortification represents a promising strategy for improving food nutritional value.

Bananas serve both as a fruit and a staple crop and are rich in L-DOPA, a key precursor for betalain biosynthesis [21], making them an ideal candidate for metabolic engineering of betalain pathways. While anthocyanins are present in the pseudostem and leaves of most banana germplasm, giving rise to red- and black-purple spots, no natural germplasm has been reported to accumulate red-purple pigmentation in the pulp.

In this study, betalains were successfully synthesized in banana pulp using the RUBY system, resulting in the development of red-purple banana fruit. This not only enhances the nutritional quality of bananas but also broadens the color palette of banana cultivars. Cavendish bananas currently account for approximately half of the global banana production. With rising consumer demand for health-enhancing and visually appealing foods, developing colorful and nutritionally enriched banana varieties will be a significant trend. The generation of betalain-rich banana seedlings and fruit in this study represents an important step toward this goal.

### 3.2. High-Efficiency Accumulation of Betalains in Transgenic Banana

Synthetic biology approaches for increasing betalain content are valuable for improving crop appearance and nutritional quality. Co-expression of BvCYP76AD1, BvDODA, and MjcDOPA5GT in various crops, such as eggplant, tomato, potato, and carrot [18], has been shown to significantly increase betalain content compared with wild-type plants. However, further improvement remains possible. In this study, betalain content in RUBY banana leaves reached 3.23 mg/g, representing a substantial increase. Two primary factors likely contributed to this elevation. Firstly, the RUBY system exhibited high expression efficiency after integration into the banana genome. In line #1, the expression levels of BvCYP76AD1, BvDODA, and MjcDOPA5GT were 18.61-, 13.53-, and 4.50-fold higher, respectively, compared with the control. Similar findings have been reported in rice (*Oryza sativa*) and tobacco (*N*. *benthamiana*) using the same expression system [15]. Secondly, bananas naturally contain abundant precursor compounds, such as tyrosine and L-DOPA, which are essential for betalain biosynthesis. The availability of these precursors likely facilitated the high accumulation of betalains in the transgenic lines.

The unpredictable negative effect on host plants has been reported in many plant metabolic engineering studies [24]. In this study, high betalain content affects the growth of banana. Our results showed that high betalain content leads to a decrease in photosynthetic efficiency, the transpiration rate, etc., and thus imposes certain restrictions on growth. Some crops, such as potato, eggplant, and tomato, also caused growth reduction by expressing betalain synthesis genes driven by a CaMV 35S constitutive promoter [18,19]. But according to the previous studies, when these three betalain synthesis genes are controlled under the specifical promoter in rice, such as globulin-1 or a DR5 promoter, they can successfully prevent them from affecting plant growth [14,17]. In tomato, betalain synthesis genes are controlled under a fruit-specific E8 promoter by restricting betalain accumulation to fruit, which does not display any apparent developmental phenotype or growth retardation. Therefore, the higher content of betalain synthesis in plant tissues may be the main reason for the negative effect on plant growth. The next step in our work will be to further study the specific promoter of banana, aiming to eliminate the negative effect on the growth of banana plants.

This RUBY method also has potential for high-value crops, such as ornamental plants and medicinal plants. However, although enriching the fruit with antioxidant compounds is needed to enhance the nutritional value for human health, transgenic plants are not yet acceptable in various countries. In the short term, using banana pulp, plants and ECSs as bioreactors to separate betalains and applying this method in industrial production might be a good strategy. But in the long term, more works need to be carried out before offering such transgenic bananas to the consumers.

### 3.3. RUBY Serves as a Visual Screening Marker for Banana Genetic Transformation

Currently, Agrobacterium-mediated ECS transformation is the primary method for banana genetic modification. A major bottleneck in this process is the liquid-phase resistance screening step, which typically requires three to four rounds of selection and takes more than 45 days. Positive samples during this period are most often confirmed through GUS staining [25,26,27] or fluorescence-based assays [28]. In the present study, red-purple coloration was visibly observed in transformed cell clusters after just one round of liquid resistance screening, allowing for direct identification and selection for somatic embryo induction. This reduced screening time, improved blastocyst formation rates, and enhanced the efficiency of somatic embryo germination. In the future application of banana genetic transformation, this RUBY system can be used for gene modification, such as disease-resistant molecular breeding. Therefore, the RUBY system can serve as an effective visual marker for banana transformation, offering both functional and practical advantages in genetic engineering workflows.

## 4. Materials and Methods

### 4.1. Plant Materials and Vectors

Embryogenic cell suspensions (ECSs) of *Musa acuminata* Cavendish banana cultivar (*Musa* spp., AAA group cv. “Brazilian”) were derived from immature male flowers and cultured at 25 ± 2 °C in the dark, as previously described [29]. ECSs were subcultured every two weeks in M2 medium, consisting of MS medium supplemented with 1 mg/L 2,4-D, 1 mg/L biotin, 100 mg/L glutamine, 100 mg/L malt extract, and 45 g/L sucrose (pH 5.3). Whole banana plants were grown in a temperature-controlled greenhouse under a 14 h light/10 h dark photoperiod at 28 °C and 40% relative humidity.

The RUBY expression vector pYL1300H-CDG was kindly provided by Professor Zhu Qinlong (South China Agricultural University). Three key betalain biosynthesis genes—BvCYP76AD1S (GenBank: ON600884.1), BvDODA1S (GenBank: ON600882.1), and cDOPA5GT (GenBank: ON600883.1)—were cloned into the binary vector pYL1300H-UNiE under the control of the CaMV 35S promoter to generate the RUBY plasmid (Figure 1).

### 4.2. Banana Transformation

Genetic transformation of banana ECSs was performed using *Agrobacterium* tumefaciens, based on a modified protocol [29], as outlined below.

#### 4.2.1. Preparation of Infection Solution

The RUBY plasmid or empty vector (EV) was transformed into the A. tumefaciens strain EHA105. Positive colonies were cultured in LB solid medium containing 50 mg/L kanamycin sulfate and 25 mg/L rifampicin at 28 °C and 180× *g* until an OD_600_ of 0.8 was reached. Cultures were centrifuged at 5000× *g* for 5 min, and the bacterial pellet was resuspended in 40 mL of M2 medium to prepare the infection solution.

#### 4.2.2. Infection and Selection of ECSs

ECSs cultured for 10 days were incubated in 40 mL of the infection solution at 27 °C in the dark for 2 h. Subsequently, co-cultivation was carried out on a shaker at 50× *g* for 1 week. After co-cultivation, ECSs were washed three times with pulp M2 medium containing 500 mg/L cefotaxime sodium. Selection was conducted in 40 mL liquid M2 medium supplemented with 5 mg/L hygromycin at 27 °C and 100× *g*.

#### 4.2.3. Plantlet Regeneration

Red-purple cell masses were selected during the screening process and transferred to an embryo induction medium in the dark. Following the development of mature embryos, germination was induced under a 12 h light/12 h dark cycle until the seedlings reached 2 cm in height. The seedlings were then transferred to a rooting medium. Plants transformed with the empty vector served as controls.

### 4.3. Transient Gene Expression in N. benthamiana Leaves and Banana Fruits

Transient expression in *N. benthamiana* was conducted following a modified protocol [30]. The RUBY or EV constructs were transformed into *A*. *tumefaciens* EHA105, cultured in liquid LB at 28 °C for 8 h, and harvested by centrifugation. Cells were resuspended in an infiltration buffer containing 10 mM 2-(4-Morpholino) ethanesulfonic acid, 10 mM MgCl_2_, and 200 μM acetosyringone (pH 5.6). The OD_600_ was adjusted to 0.6 and incubated at room temperature for 2–3 h prior to leaf infiltration in 4-week-old *N. benthamiana* plants. Pigment accumulation was monitored over 3 days post-infiltration.

**Figure 4:**
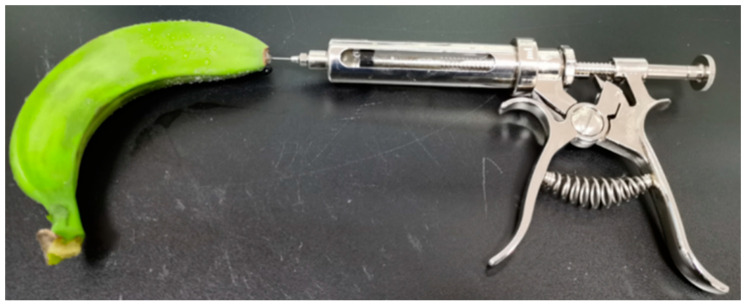
Transient expression in banana fruits.

For banana fruits, transient transformation was carried out using a modified version of the method described by Shan et al. [31]. As shown in Figure 4, young banana fruits were slowly injected with 1 mL, 2 mL, 3 mL, and 4 mL of the infiltration solution using a veterinary metal syringe. Treated fruits were incubated at 27 °C and 75% humidity for 4 days before evaluation. Fruits and leaves infiltrated with the empty vector served as negative controls.

### 4.4. Extraction and Quantification of Betalains

Approximately 0.5 g of frozen tissue was ground with liquid nitrogen and mixed with 5 mL of 80% methanol. The mixture was sonicated for 5 min (SB25-12DT, Ningbo, China), incubated at 25 °C in the dark for 20 min, and centrifuged at 10,000× *g* for 5 min. The supernatant was filtered through a 0.22 µm PVDF membrane. Betalain content was determined by measuring absorbance at 538 nm using a spectrophotometer (Tecan Biotech Co., Ltd., Männedorf, Switzerland). The concentration was calculated using the following formula:

Content (mg/100 g) = (A × DF × W × V × 100)/(S × P × L)

where A = absorbance, DF = dilution factor, W = molecular weight of betalains (550 g/mol), V = volume (mL), S = molar extinction coefficient (60,000 L/mol·cm), P = sample weight (g), and L = length of the cuvette (1 cm).

### 4.5. RNA Isolation and RT-qPCR Analysis

Total RNA was extracted from banana leaves using an RNA Out Kit (Tiandz Biotech Co., Ltd., Beijing, China), following the manufacturer’s instructions. Reverse transcription and qPCR were performed using an Evo M-MLV One Step RT-qPCR Kit (Accurate Biotech Co., Ltd., Changsha, China) and ChamQ Universal SYBR qPCR Master Mix (Vazyme Biotech Co., Ltd., Nanjing, China) on a StepOne real-time PCR system (Applied Biosystems, Waltham, MA, USA).

MaActin was used as the endogenous reference gene. Gene expression was calculated using the 2^−∆∆CT^ method. Each sample was analyzed in three biological replicates, with three technical replicates each. All primers are listed in Table 1.

### 4.6. PCR Identification of Transgenic Banana Plants

Genomic DNA was extracted from red-purple transgenic banana leaves using the CTAB (cetyltrimethylammonium bromide) method and used as the template for PCR. Specific primers were used to identify the transgenes:

BvCYP76AD1S (product: 1491 bp):

F: 5′-ATGGATCATGCCACACTCGC-3′;

R: 5′-GTACCTTGGAATCGGGATGAGC-3′.

BvDODA1S (product: 825 bp):

F: 5′-ATGAAGATGATGAACGGCGAGG-3′;

R: 5′-GGCGGATGTAAACTTGTAGGAGC-3′.

cDOPA5GT (product: 1511 bp):

F: 5′-ATGACCGCCATCAAGATGAACA-3′;

R: 5′-GGCGCGCCTCACTGGA-3′.

### 4.7. Liquid Chromatography–Mass Spectrometry (LC-MS)

The LC-MS analysis was performed using a DIONEX Ultimate 3000 UHPLC system (Agilent, Waltham, MA, USA) equipped with an electrospray ionization. Chromatographic separation conditions were performed on a C18 reversed-phase column (1.8 μm, 2.1 mm × 100 mm) with a solvent flow rate of 1 mL min^−1^ at a column temperature of 25 °C. The betalain extract samples were treated with 0.1% formic acid in water (solvent A, *v*/*v*) and 100% methanol (solvent B) mixture. The elution gradient programs were as follows: 0–20 min, 10% solvent B; 20–30 min, 30% solvent B; 30–35 min, 100% solvent B. The sample injection volume of all extract was set at 20 µL. Compound identification was confirmed by comparing their UV and spectra with standard samples.

### 4.8. Statistical Analysis

GraphPad Prism 8.0 software was used for data analysis and graphing. Statistical significance of two groups was evaluated by Student’s *t*-test (* *p* < 0.05, ** *p* < 0.01, *** *p* < 0.001). Statistical significance of three or more groups was evaluated by analysis of variance (ANOVA), and different letters above the columns indicated a significant difference.

## 5. Conclusions

This study developed a new method of banana transformation, including stable and transient genetic transformations. Transient transformation by injecting 3 mL of *Agrobacterium* suspension into immature fruits produced betalains in pulps up to 1.78 mg/g. In stable transformation, embryonic cell suspensions expressing RUBY exhibited a red-purple coloration after the first screening, reducing the selection period from 45 to 15 days. Betalain accumulation in RUBY transgenic leaf can reach 3.23 mg/g. Therefore, the RUBY system can be used for efficient banana transformation and for the development of betalain-rich *Musa* germplasm.

## Figures and Tables

**Table 1:** RT-qPCR primers used in this study.

Primer Name	Sequences (5′-3′)	Notes
*BvCYP76AD1S*-F	CTGCGAGATCGATGTTAAGG	RT-qPCR primers of BvCYP76AD1S gene
*BvCYP76AD1S*-R	GAACTTCTCGTCCATGTCGAG	
*BvDODA1S*-F	CACCGCACTACTTCGATGGTG	RT-qPCR primers of BvDODA1S gene
*BvDODA1S*-R	GAGTGGATAAGCTCGGCCTTC	
*cDOPA5GT*-F	CATCGTCGAGATGATCCTTGAG	RT-qPCR primers of cDOPA5GT gene
*cDOPA5GT*-R	GAAATCGTCGATCGCCTTCACG	
*MaActin*-F	ACATTGTCAGGTGGGGAGTT	RT-qPCR primers of endogenous reference gene *MaActin*
*MaActin*-R	CCTTTTGTTCCACACGAGATT	

## Data Availability

Data are contained within this article.
